# Supplementary material for: Carabid community structure in northern China grassland ecosystems: Effects of local habitat on species richness, species composition and functional diversity
Source: PeerJ. 2019 Jan 9;6:e6197. doi: 10.7717/peerj.6197 (PMC6330033; doi:10.7717/peerj.6197)
Supplement: Supplemental Information 1 — PB: Plant dry biomass, PC: Plant cover, PD: Plant density, PH: Plant height, PSD: Plant species diversity (richness); SBD: Soil bulk density, SL: Soil litter, SM: Soil moisture, ST: Soil temperature; Hum: Humidity, Prec: Precipitation, Temp: Temperature. [file peerj-07-6197-s001.docx]

|  |  | Regional scale | | Grassland types | | | | | |
| --- | --- | --- | --- | --- | --- | --- | --- | --- | --- |
|  |  |  |  | Desert Steppe | | Typical Steppe | | Meadow Steppe | |
|  |  | Range | Mean ± SD | Range | Mean ± SD | Range | Mean ± SD | Range | Mean ± SD |
| Vegetation | PB | 21.84 - 296.96 | 91.9 ± 55.07 | 21.84 - 122.24 | 54.0 ± 17.82 | 30.00 - 296.96 | 108.2 ± 63.50 | 23.20 - 209.92 | 86.5 ± 42.06 |
|  | PC | 8.00 - 93.00 | 52.3 ± 17.61 | 8.00 - 58.00 | 32.8 ± 12.43 | 25.00 - 93.00 | 42.6 ± 16.83 | 25.00 - 79.00 | 50.3 ± 12.06 |
|  | PD | 30.00 - 153.00 | 74.6 ± 26.10 | 30.00 - 145.00 | 76.9 ± 21.62 | 30.00 - 153.00 | 85.6 ± 28.01 | 30.00 - 85.00 | 57.0 ± 11.95 |
|  | PH | 5.00 - 85.00 | 37.6 ± 17.65 | 5.00 - 52.00 | 30.6 ± 12.97 | 11.00 - 85.00 | 60.1 ± 18.58 | 12.00 - 78.00 | 33.5 ± 15.98 |
|  | PSD | 1.00 - 10.00 | 5.7 ± 1.80 | 1.00 - 7.00 | 4.4 ± 1.65 | 2.00 - 9.00 | 5.6 ± 1.64 | 3.00 - 10.00 | 6.6 ± 1.64 |
| Soil | SBD | 0.75 - 2.28 | 1.2 ± 0.17 | 1.23 - 1.56 | 1.4 ± 0.07 | 0.89 - 1.51 | 1.2 ± 0.12 | 0.75 - 2.28 | 1.1 ± 0.17 |
|  | SL | 20.88 - 171.56 | 59.3 ± 27.55 | 26.48 - 78.28 | 46.18 ± 11.71 | 21.40 - 171.56 | 67.9 ± 34.00 | 20.88 - 96.60 | 52.9 ± 15.55 |
|  | SM | 0.01 - 0.42 | 0.2 ± 0.08 | 0.01 - 0.07 | 0.04 ± 0.01 | 0.06 - 0.30 | 0.2 ± 0.04 | 0.06 - 0.42 | 0.2 ± 0.07 |
|  | ST | 2.18 - 30.06 | 17.6 ± 4.96 | 17.25 - 30.06 | 23.9 ± 3.57 | 10.06 - 24.37 | 17.9 ± 2.88 | 2.18 - 21.87 | 13.8 ± 4.60 |
| Climate | Hum | 35.74 - 68.68 | 53.2 ± 11.03 | 35.74 - 64.29 | 53.1 ± 11.30 | 39.39 - 64.10 | 52.0 ± 11.08 | 36.55 - 68.68 | 53.7 ± 13.18 |
|  | Prec | 0.57 - 6.96 | 2.4 ± 1.41 | 0.57 - 3.95 | 1.9 ± 1.41 | 0.92 - 6.14 | 2.5 ± 2.07 | 0.59 - 6.96 | 2.8 ± 2.5 |
|  | Temp | 14.69 - 24.82 | 18.7 ± 3.13 | 17.66 - 24.82 | 20.4 ± 2.96 | 14.69 - 22.57 | 17.6 ± 3.25 | 15.23 - 22.41 | 17.9 ± 2.98 |
