# Supplementary material for: Carabid community structure in northern China grassland ecosystems: Effects of local habitat on species richness, species composition and functional diversity
Source: PeerJ. 2019 Jan 9;6:e6197. doi: 10.7717/peerj.6197 (PMC6330033; doi:10.7717/peerj.6197)
Supplement: Supplemental Information 2 — PB: Plant dry biomass, PC: Plant cover, PD: Plant density, PH: Plant height, PSD: Plant species diversity (richness); SBD: Soil bulk density, SL: Soil litter, SM: Soil moisture, ST: Soil temperature; Hum: Humidity, Prec: Precipitation, Temp: Temperature. [file peerj-07-6197-s002.docx]

|  |  | Regional scale | Grassland types | | |
| --- | --- | --- | --- | --- | --- |
|  |  |  | Desert Steppe | Typical Steppe | Meadow Steppe |
| Vegetation | PB | 1.97 | 1.25 | 3.18 | 1.91 |
|  | PC | 2.94 | 3.84 | 2.84 | 1.79 |
|  | PD | 1.38 | 3.03 | 1.75 | 1.80 |
|  | PH | 2.61 | 2.74 | 3.33 | 2.72 |
|  | PSD | 1.42 | 2.02 | 1.61 | 1.56 |
| Soil | SBD | 1.45 | 1.36 | 1.48 | 2.09 |
|  | SL | 2.22 | 1.46 | 3.49 | 1.28 |
|  | SM | 2.54 | 1.71 | 1.49 | 1.96 |
|  | ST | 2.90 | 9.98 | 1.62 | 3.29 |
| Climate | Hum | 2.24 | 3.75 | 3.95 | 2.78 |
|  | Prec | 2.05 | 8.93 | 2.12 | 2.82 |
|  | Temp | 2.08 | 3.69 | 2.88 | 1.99 |
