# Supplementary material for: Carabid community structure in northern China grassland ecosystems: Effects of local habitat on species richness, species composition and functional diversity
Source: PeerJ. 2019 Jan 9;6:e6197. doi: 10.7717/peerj.6197 (PMC6330033; doi:10.7717/peerj.6197)
Supplement: Supplemental Information 3 — PB: Plant dry biomass, PC: Plant cover, PD: Plant density, PH: Plant height, PSD: Plant species diversity (richness); SBD: Soil bulk density, SL: Soil litter, SM: Soil moisture, ST: Soil temperature; Hum: Humidity, Prec: Precipitation, Temp: Temperature. [file peerj-07-6197-s003.docx]

|  |  | Regional scale | Grassland types | | |
| --- | --- | --- | --- | --- | --- |
|  |  |  | Desert Steppe | Typical Steppe | Meadow Steppe |
| Vegetation | PB | 1.82 | 1.82 | 2.53 | 1.81 |
|  | PC | 2.85 | 2.00 | 3.18 | 1.41 |
|  | PD | 1.84 | 3.84 | 2.23 | 1.67 |
|  | PH | 2.83 | 2.17 | 3.23 | 2.02 |
|  | PSD | 1.19 | 2.26 | 1.56 | 1.63 |
| Soil | SBD | 1.31 | 1.57 | 1.77 | 2.47 |
|  | SL | 2.73 | 2.14 | 3.85 | 2.00 |
|  | SM | 2.18 | 2.20 | 1.32 | 2.18 |
|  | ST | 2.28 | 9.52 | 1.49 | 2.77 |
| Climate | Hum | 3.35 | 3.07 | 2.65 | 4.93 |
|  | Prec | 3.44 | 6.54 | 5.76 | 4.43 |
|  | Temp | 1.91 | 8.73 | 4.16 | 1.84 |
