# Supplementary material for: Carabid community structure in northern China grassland ecosystems: Effects of local habitat on species richness, species composition and functional diversity
Source: PeerJ. 2019 Jan 9;6:e6197. doi: 10.7717/peerj.6197 (PMC6330033; doi:10.7717/peerj.6197)
Supplement: Supplemental Information 4 [file peerj-07-6197-s004.docx]

|  | Regional scale | | Grassland types | | | | | |
| --- | --- | --- | --- | --- | --- | --- | --- | --- |
|  |  |  | Desert Steppe | | Typical Steppe | | Meadow Steppe | |
|  | Range | Mean ± SD | Range | Mean ± SD | Range | Mean ± SD | Range | Mean ± SD |
| RR | 0.00 - 16.84 | 3.41±2.90 | 0.00 - 6.00 | 1.05±1.40 | 0.00 - 16.84 | 3.87±2.83 | 0.00 - 14.75 | 3.33±2.99 |
| FD-Total | 0.00 - 0.34 | 0.12±0.10 | 0.00 - 0.34 | 0.04±0.10 | 0.00 - 0.34 | 0.14±0.10 | 0.00 - 0.33 | 0.12±0.10 |
| FD-Movement | 0.00 - 0.39 | 0.12±0.12 | 0.00 - 0.39 | 0.04±0.10 | 0.00 - 0.36 | 0.15±0.12 | 0.00 - 0.35 | 0.13±0.11 |
| FD-Size | 0.00 - 0.41 | 0.12±0.11 | 0.00 - 0.41 | 0.05±0.11 | 0.00 - 0.33 | 0.14±0.10 | 0.00 - 0.37 | 0.12±0.10 |
