# Supplementary material for: Carabid community structure in northern China grassland ecosystems: Effects of local habitat on species richness, species composition and functional diversity
Source: PeerJ. 2019 Jan 9;6:e6197. doi: 10.7717/peerj.6197 (PMC6330033; doi:10.7717/peerj.6197)
Supplement: Supplemental Information 5 [file peerj-07-6197-s005.docx]

|  |  | Rarefied richness | FD-Total | FD-Movement | FD-Size |
| --- | --- | --- | --- | --- | --- |
|  |  |  |  |  |  |
| Effect of grasslands and model characteristics | r^2^ | 0.23 | 0.16 | 0.15 | 0.12 |
|  | logLik | -362.73 | 477.25 | 147.77 | 277.84 |
|  | AIC | 745.46 | -934.50 | -275.54 | -535.68 |
|  | BIC | 786.49 | -893.47 | -234.52 | -494.76 |
|  | F-value (P-value) | **45.90 (0.006)** | **15.69 (0.026)** | **29.14 (0.011)** | **24.98 (0.014)** |
| Post hoc Tukey test | DesertSteppe - MeadowSteppe | **0.71 ± 0.08 (<0.0001)** | **0.07 ± 0.03 (0.049)** | **0.14 ± 0.06 (0.039)** | **0.10 ± 0.04 (0.032)** |
|  | DesertSteppe - TypicalSteppe | **0.72 ± 0.14 (<0.0001)** | **0.09 ± 0.02 (<0.0001)** | **0.17 ± 0.02 (<0.0001)** | **0.12 ± 0.02 (<0.0001)** |
|  | MeadowSteppe - TypicalSteppe | 0.02 ± 0.13 (0.990) | 0.02 ± 0.03 (0.774) | 0.03 ± 0.06 (0.856) | 0.02 ± 0.04 (0.771) |
