# Supplementary material for: Carabid community structure in northern China grassland ecosystems: Effects of local habitat on species richness, species composition and functional diversity
Source: PeerJ. 2019 Jan 9;6:e6197. doi: 10.7717/peerj.6197 (PMC6330033; doi:10.7717/peerj.6197)
Supplement: Supplemental Information 6 — Percentages of variance explained and P-values are given in parentheses. PB: Plant dry biomass, PC: Plant cover, PD: Plant density, PH: Plant height, PSD: Plant species diversity (richness); SBD: Soil bulk density, SL: Soil litter, SM: Soil moisture, ST: Soil temperature; Hum: Humidity, Prec: Precipitation, Temp: Temperature. [file peerj-07-6197-s006.docx]

|  | Regional scale | Grassland types | | |
| --- | --- | --- | --- | --- |
| Eigenvalues |  | Desert Steppe | Typical Steppe | Meadow Steppe |
| Total constrained (and proportion %) | 1.86 (23.66) | 0.54 (12.77) | 1.15(35.31) | 0.92(20.63) |
| CCA1 | 0.58 (31.43; 0.001) | 0.38 (70.18; 0.006) | 0.55(48.25; 0.001) | 0.47(50.10; 0.001) |
| CCA2 | 0.48 (25.63; 0.001) | 0.16 (29.82; 0.117) | 0.25(21.68; 0.001) | 0.21(23.12; 0.001) |
| Variables included in the model | PB; PC PD; PH; PSD; SBD; SL; SM; ST; Hum; Prec; Temp | Hum; Temp | PB; PC; PD; SL; Hum; Prec; Temp | PB; PH; SL; Hum; Temp |
