# Supplementary material for: Carabid community structure in northern China grassland ecosystems: Effects of local habitat on species richness, species composition and functional diversity
Source: PeerJ. 2019 Jan 9;6:e6197. doi: 10.7717/peerj.6197 (PMC6330033; doi:10.7717/peerj.6197)
Supplement: Supplemental Information 7 — Model characteristics: r2 = adjusted coefficient of determination, rlogLik = restricted log-likehood, AIC = Akaike Information Criterion, BIC = Bayesian Information Criterion. Parameter estimated coefficients (± standard error) and P-values (in parentheses) are given for each predictor. Significant effects are in bold. Predictors abbreviations: PB: Plant dry biomass, PC: Plant cover, PD: Plant density, PH: Plant height, PSD: Plant species diversity (richness); SBD: Soil bulk density, SL: Soil litter, SM: Soil moisture, ST: Soil temperature; Hum: Humidity, Prec: Precipitation, Temp: Temperature. [file peerj-07-6197-s007.docx]

|  |  | Regional scale | Grassland types | | |
| --- | --- | --- | --- | --- | --- |
|  |  |  | Desert steppe | Typical steppe | Meadow steppe |
| Model characteristics | r^2^ | 0.30 | 0.21 | 0.28 | 0.41 |
|  | rlogLik | 356.18 | 42.08 | 158.86 | 114.91 |
|  | AIC | -678.35 | -50.17 | -283.73 | -195.82 |
|  | BIC | -608.50 | -10.77 | -225.65 | -144.64 |
| Vegetation | PB | **-0.02 ± 0.01 (0.002)** | 0.01 ± 0.01 (0.225) | -0.00 ± 0.01 (0.829) | **-0.02 ± 0.01 (0.039)** |
|  | PC | -0.01 ± 0.01 (0.111) | 0.01 ± 0.02 (0.526) | 0.01 ± 0.01 (0.449) | **-0.02 ± 0.01 (0.017)** |
|  | PD | -0.00 ± 0.01 (0.563) | -0.03 ± 0.02 (0.080) | 0.01 ± 0.01 (0.162) | 0.01 ± 0.01 (0.326) |
|  | PH | 0.01 ± 0.01 (0.149) | 0.01 ± 0.02 (0.682) | 0.00 ± 0.01 (0.908) | 0.02 ± 0.01 (0.070) |
|  | PSD | 0.01 ± 0.01 (0.386) | -0.01 ± 0.01 (0.288) | -0.00 ± 0.01 (0.555) | 0.01 ± 0.01 (0.094) |
| Soil | SBD | -0.01 ± 0.01 (0.386) | 0.01 ± 0.01 (0.302) | -0.01 ± 0.01 (0.270) | -0.00 ± 0.01 (0.663) |
|  | SL | 0.00 ± 0.01 (0.528) | 0.02 ± 0.01 (0.158) | -0.01 ± 0.01 (0.479) | 0.01 ± 0.01 (0.297) |
|  | SM | -0.01 ± 0.01 (0.140) | 0.01 ± 0.01 (0.365) | -0.00 ± 0.01 (0.710) | 0.01 ± 0.01 (0.450) |
|  | ST | **-0.02 ± 0.01 (0.013)** | -0.01 ± 0.03 (0.790) | **-0.02 ± 0.01 (0.021)** | **0.03 ± 0.01 (0.031)** |
| Climate | Hum | 0.01 ± 0.01 (0.125) | **0.05 ± 0.02 (0.016)** | **0.03 ± 0.01 (0.025)** | -0.01 ± 0.01 (0.486) |
|  | Prec | **0.02 ± 0.01 (0.002)** | -0.05 ± 0.03 (0.065) | 0.02 ± 0.01 (0.051) | **0.02 ± 0.01 (0.049)** |
|  | Temp | **0.03 ± 0.01 (<0.0001)** | 0.02 ± 0.02 (0.411) | **0.06 ± 0.01 (<0.0001)** | 0.02 ± 0.01 (0.062) |
|  | Intercept | **0.12 ± 0.00 (<0.000)** | **0.04 ± 0.01 (<0.0001)** | **0.15 ± 0.01 (<0.0001)** | **0.13 ± 0.01 (<0.0001)** |
