# Supplementary material for: Carabid community structure in northern China grassland ecosystems: Effects of local habitat on species richness, species composition and functional diversity
Source: PeerJ. 2019 Jan 9;6:e6197. doi: 10.7717/peerj.6197 (PMC6330033; doi:10.7717/peerj.6197)
Supplement: Supplemental Information 8 — Model characteristics: r2 = adjusted coefficient of determination, rlogLik = restricted log-likehood, AIC = Akaike Information Criterion, BIC = Bayesian Information Criterion. Parameter estimated coefficients (± standard error) and P-values (in parentheses) are given for each predictor. Significant effects are in bold. Predictors abbreviations: PB: Plant dry biomass, PC: Plant cover, PD: Plant density, PH: Plant height, PSD: Plant species diversity (richness); SBD: Soil bulk density, SL: Soil litter, SM: Soil moisture, ST: Soil temperature; Hum: Humidity, Prec: Precipitation, Temp: Temperature. [file peerj-07-6197-s008.docx]

|  |  | Regional scale | Grassland types | | |
| --- | --- | --- | --- | --- | --- |
|  |  |  | Desert steppe | Typical steppe | Meadow steppe |
| Model characteristics | r^2^ | 0.26 | 0.23 | 0.22 | 0.34 |
|  | rlogLik | 385.98 | 33.86 | 184.31 | 125.80 |
|  | AIC | -737.95 | -33.73 | -334.63 | -217.60 |
|  | BIC | -668.09 | 5.67 | -276.55 | -166.42 |
| Vegetation | PB | -0.01 ± 0.01 (0.082) | 0.00 ± 0.01 (0.765) | 0.00 ± 0.01 (0.845) | -0.01 ± 0.01 (0.424) |
|  | PC | -0.01 ± 0.01 (0.100) | 0.01 ± 0.02 (0.600) | 0.00 ± 0.01 (0.873) | **-0.02 ± 0.01 (0.040)** |
|  | PD | -0.01 ± 0.01 (0.123) | -0.04 ± 0.02 (0.056) | 0.00 ± 0.01 (0.859) | 0.01 ± 0.01 (0.084) |
|  | PH | 0.01 ± 0.01 (0.103) | 0.00 ± 0.02 (0.839) | 0.00 ± 0.01 (0.976) | **-0.03 ± 0.01 (0.014)** |
|  | PSD | 0.00 ± 0.01 (0.595) | -0.01 ± 0.02 (0.696) | -0.01 ± 0.01 (0.376) | 0.00 ± 0.01 (0.615) |
| Soil | SBD | -0.01 ± 0.01 (0.315) | 0.01 ± 0.01 (0.358) | -0.01 ± 0.01 (0.384) | -0.00 ± 0.01 (0.877) |
|  | SL | -0.00 ± 0.01 (0.529) | 0.02 ± 0.01 (0.181) | -0.01 ± 0.01 (0.212) | 0.00 ± 0.01 (0.796) |
|  | SM | -0.01 ± 0.01 (0.261) | 0.01 ± 0.01 (0.417) | -0.00 ± 0.01 (0.859) | 0.01 ± 0.01 (0.530) |
|  | ST | **-0.02 ± 0.01 (0.016)** | -0.01 ± 0.03 (0.730) | -0.01 ± 0.01 (0.121) | 0.02 ± 0.01 (0.070) |
| Climate | Hum | **0.02 ± 0.01 (0.004)** | **0.06 ± 0.02 (0.009)** | **0.03 ± 0.01 (0.003)** | -0.00 ± 0.01 (0.730) |
|  | Prec | **0.01 ± 0.01 (0.025)** | -0.06 ± 0.03 (0.077) | 0.01 ± 0.01 (0.255) | 0.02 ± 0.01 (0.060) |
|  | Temp | **0.03 ± 0.01 (<0.0001)** | 0.02 ± 0.02 (0.449) | **0.05 ± 0.01 (<0.0001)** | **0.03 ± 0.01 (0.006)** |
|  | Intercept | **0.12 ± 0.00 (<0.0001)** | **0.05 ± 0.01 (<0.0001)** | **0.14 ± 0.01 (<0.0001)** | **0.12 ± 0.01 (<0.0001)** |
